# Integrated morphological, metabolome, and transcriptome analyses revealed the mechanism of exogenous gibberellin promoting petiole elongation in *Oenanthe javanica*

**DOI:** 10.3389/fpls.2023.1225635

**Published:** 2023-07-17

**Authors:** Kai Feng, Xibei Li, Yajie Yan, Ruozhenyi Liu, Zixuan Li, Nan Sun, Zhiyuan Yang, Shuping Zhao, Peng Wu, Liangjun Li

**Affiliations:** ^1^ College of Horticulture and Landscape Architecture, Yangzhou University, Yangzhou, China; ^2^ Joint International Research Laboratory of Agriculture and Agri−Product Safety of Ministry of Education of China, Yangzhou University, Yangzhou, China

**Keywords:** water dropwort, metabolites, cellulose synthase, transcription, gene

## Abstract

*Oenanthe javanica* (Blume) DC. is a popular vegetable with unique flavor and its leaf is the main product organ. Gibberellin (GA) is an important plant hormone that plays vital roles in regulating the growth of plants. In this study, the plants of water dropwort were treated with different concentrations of GA_3_. The plant height of water dropwort was significantly increased after GA_3_ treatment. Anatomical structure analysis indicated that the cell length of water dropwort was elongated under exogenous application of GA_3_. The metabolome analysis showed flavonoids were the most abundant metabolites and the biosynthesis of secondary metabolites were also regulated by GA_3_. The exogenous application of GA_3_ altered the gene expressions of plant hormone signal transduction (*GID* and *DELLA*) and metabolites biosynthesis pathways to regulate the growth of water dropwort. The GA contents were modulated by up-regulating the expression of GA metabolism gene *GA2ox*. The differentially expressed genes related to cell wall formation were significantly enriched. A total of 22 cellulose synthase involved in cellulose biosynthesis were identified from the genome of water dropwort. Our results indicated that GA treatment promoted the cell elongation by inducing the expression of cellulose synthase and cell wall formation in water dropwort. These results revealed the molecular mechanism of GA-mediated cell elongation, which will provide valuable reference for using GA to regulate the growth of water dropwort.

## 1 Introduction

Water dropwort [*Oenanthe javanica* (Blume) DC.] is a perennial aquatic herb in the Apiaceae family, which is mainly grown in tropical and temperate regions (Lu and Li, 2019). Water dropwort contains various bioactive substances and it is well known to have many medicinal effects, such as promoting digestion, reducing blood pressure and blood sugar (Yang et al., 2014; Kim et al., 2016). Water dropwort is also consumed as a popular vegetable because it is rich in vitamins, proteins, dietary fibers, and flavonoids (Feng et al., 2018). Fresh stems and petioles are the main edible parts of water dropwort, which has a unique aroma and taste (Park et al., 2015; Feng et al., 2022). The yield of water dropwort was closely related to the petiole length; thus, GA is an effective way to improve the petiole length in the production of water dropwort.

Gibberellins (GAs) are important plant hormones derived from the tetracyclic backbone of diterpenic acids, which have multiple functions in regulating various developmental processes and environmental responses in plants (Sponsel, 2016). The biosynthesis and signal transduction pathway of GAs in plants has been extensively presented (Hedden, 2020). The geranylgeranyl diphosphate (GGPP) was converted into *ent*-kaurene catalyzed by ent-copalyl diphosphate synthase (CPS) and *ent*-kaurene synthase (KS). GA is then synthesized from *ent*-kaurene by various enzymes in plants, including *ent*-kaurene oxidase (KO), *ent*-kaurenoic acid oxidase (KAO), GA20-oxidase (GA20ox) and GA3-oxidase (GA3ox). The inactivation of bioactive GAs was regulated by the GA2-oxidase (GA2ox). The signal transduction of GAs involves many proteins, such as GIBBERELLIN INSENSITIVE DWARF1 (GID1), DELLA, specific ubiquitin E3 ligase complex (SCFSLY1/GID2/SNE), and SLEEPY1 (SLY1) (Yamaguchi, 2008).

In plants, GAs play vital roles in many biological processes (Daviere and Achard, 2013). Specially, GAs regulate the plant height by promoting the stem elongation (Ohtaka et al., 2020; Li et al., 2023). Altering GA levels by exogenous application or genetic approaches was widely used to promote yields and productivity in crops production (Gao and Chu, 2020). The semidwarf varieties derived from GA biosynthetic genes *semidwarf1* (*GA20ox2*) altered the GAs contents and resulted in the high-yielding performance in rice production, which was extensively applied in the 20th century’s Green Revolution (Spielmeyer et al., 2002). In addition, exogenous application of GAs was also an effective method for regulating the growth of plants (Pearce et al., 2013; Cai et al., 2018). Previous study indicated that the height and biomass of *Neolamarckia cadamba* were increased after GAs treatment (Li et al., 2022). However, the effects of GAs application on the growth and metabolite accumulation of water dropwort have not been reported and the suitable application concentration of GAs was still unknown.

In this study, the plants of water dropwort were treated with different concentrations of GA_3_ and GA inhibitor. The effects of various treatments on the growth of water dropwort were determined. The metabolomic and transcriptomic analysis were conducted to identify the metabolites and key regulatory genes in water dropwort under GAs and uniconazole treatments. This study increases our understanding for the regulatory mechanism of GA-mediated cell elongation and provides the suitable approach to regulate the height and metabolites of water dropwort.

## 2 Materials and methods

### 2.1 Plant materials and experimental treatments

The water dropwort variety ‘Fuqin No.1’ was used as plant material in this study, which was stored in the aquatic vegetable experimental base of Yangzhou University (32°39′N, 119°42′E). The water dropwort was grown in the pots (38 cm × 28 cm) containing fertile soil under natural condition. The water dropwort was irrigated with water every 5 days and the 30-day-old plants were treated with different concentrations of GA_3_. The treatments of T1, T2, T3, T4 represented 40 mg/L, 80 mg/L, 120 mg/L, 160 mg/L of GA_3_, respectively. T5 indicated the treatment of uniconazole (a gibberellin inhibitor, 50 mg/L). The water dropwort treated with distilled water was set as control group (CK). Each treatment was performed for three biological replicates. The 65-day-old plants of water dropwort were measured and the samples were harvested for further analysis.

### 2.2 Anatomical structure analysis

The petioles of water dropwort under different treatments were sampled for anatomical structure analysis. The middle part of petioles was cut into 3 mm and the tissue structure was immobilized in the phosphate buffer solution containing 2.5% of glutaraldehyde (pH = 7.2). The slices were dewaxed and dehydrated with xylene and ethanol, respectively. The samples were then stained by the safranin-*O* (1%) for 2 h and cleaned by ethanol (Que et al., 2018). The anatomical structure of water dropwort was observed by light microscope. The Image J software was used to detect the cell length of water dropwort under different treatments (Schindelin et al., 2015).

### 2.3 Determination of GAs contents

To further investigate the changes of GAs contents in water dropwort, the petioles of CK, T2, and T5 groups were collected for determination of GAs contents. The samples of water dropwort were frozen in liquid nitrogen and stored in -80 °C refrigerator. The samples were then sent to MetWare (http://www.metware.cn/) for the determination of GAs contents. The measurement was performed using AB Sciex QTRAP 6500 LC-MS/MS platform (He et al., 2020).

### 2.4 Metabolites analysis

The broadly targeted metabolomics of water dropwort were conducted in MetWare (http://www.metware.cn/). Briefly, the plant samples were first freeze-dried and crushed. Fifty mg of lyophilized powder were dissolved in methanol solution (70%). The samples were then centrifugated and filtrated for further UPLC-MS/MS analysis. Four μL of the above extraction were injected into the UPLC-ESI-MS/MS system. The effluent was further analyzed by an ESI-triple quadrupole-linear ion trap (QTRAP)-MS (Li et al., 2021). The differentially expressed metabolites (DEMs) among different groups were identified based on the variable importance in projection (VIP) and fold change (FC) values. The VIP values were obtained from the OPLS-DA model. The metabolites with a VIP value ≥ 1 and FC value ≥2 or ≤0.5 were determined as DEMs (Cao et al., 2022). The identified metabolites were annotated based on the KEGG database (http://www.kegg.jp/kegg/compound/).

### 2.5 Transcriptome analysis

The petioles of water dropwort from three biological replicates under CK, T2, and T5 treatments were sampled and frozen in liquid nitrogen for transcriptome analysis (Zhang et al., 2017). The purity and integrity of extracted total RNA was detected by NanoDrop 2000 and Agient2100/LabChip GX, respectively. The RNA-seq libraries were constructed by Biomarker Technologies Co., Ltd. (Beijing, China). The constructed libraries were sequenced by the Illumina NovaSeq6000 platform. Based on the high-quality genome of water dropwort (Liu et al., 2023), the clean reads of transcriptome were compared to the reference genome by the HISAT2 software (Kim et al., 2015). The expression levels of the gene were calculated based on the read counts and normalized by the fragments per kilobase of transcript per million fragments mapped (FPKM) method (Trapnell et al., 2010). The transcriptome analysis of water dropwort was conducted on the BMKCloud (www.biocloud.net). The raw data has been deposited in SRA database with the ID of PRJNA977200.

### 2.6 Identification of cellulose synthase family in water dropwort

In order to identify the members of the cellulose synthase (CES) family in water dropwort, the HMM model (PF03552) was used to search the CES family member from water dropwort by TBtools (Chen et al., 2020). The obtained candidate CES family sequences were further determined by analysis of conserved domains in Pfam databases (Finn et al., 2014). The sequences of Arabidopsis CES family were downloaded from The Arabidopsis Information Resource (TAIR) (Rhee et al., 2003). The protein sequences of CES family from water dropwort and Arabidopsis were used to construct the phylogenetic tree by MEGA 7.0 using neighbor-joining method (Kumar et al., 2016). The promoter sequence (upstream 2000 bp) of different *CESA* family genes were extracted from the genome of water dropwort (Liu et al., 2023). The cis-acting elements analysis of promoter was performed on the PlantCARE (Lescot et al., 2002) and the different cis-acting elements were visualized by TBtools (Chen et al., 2020).

## 3 Results

### 3.1 Growth analysis of water dropwort under GA treatment

**Figure 1:**
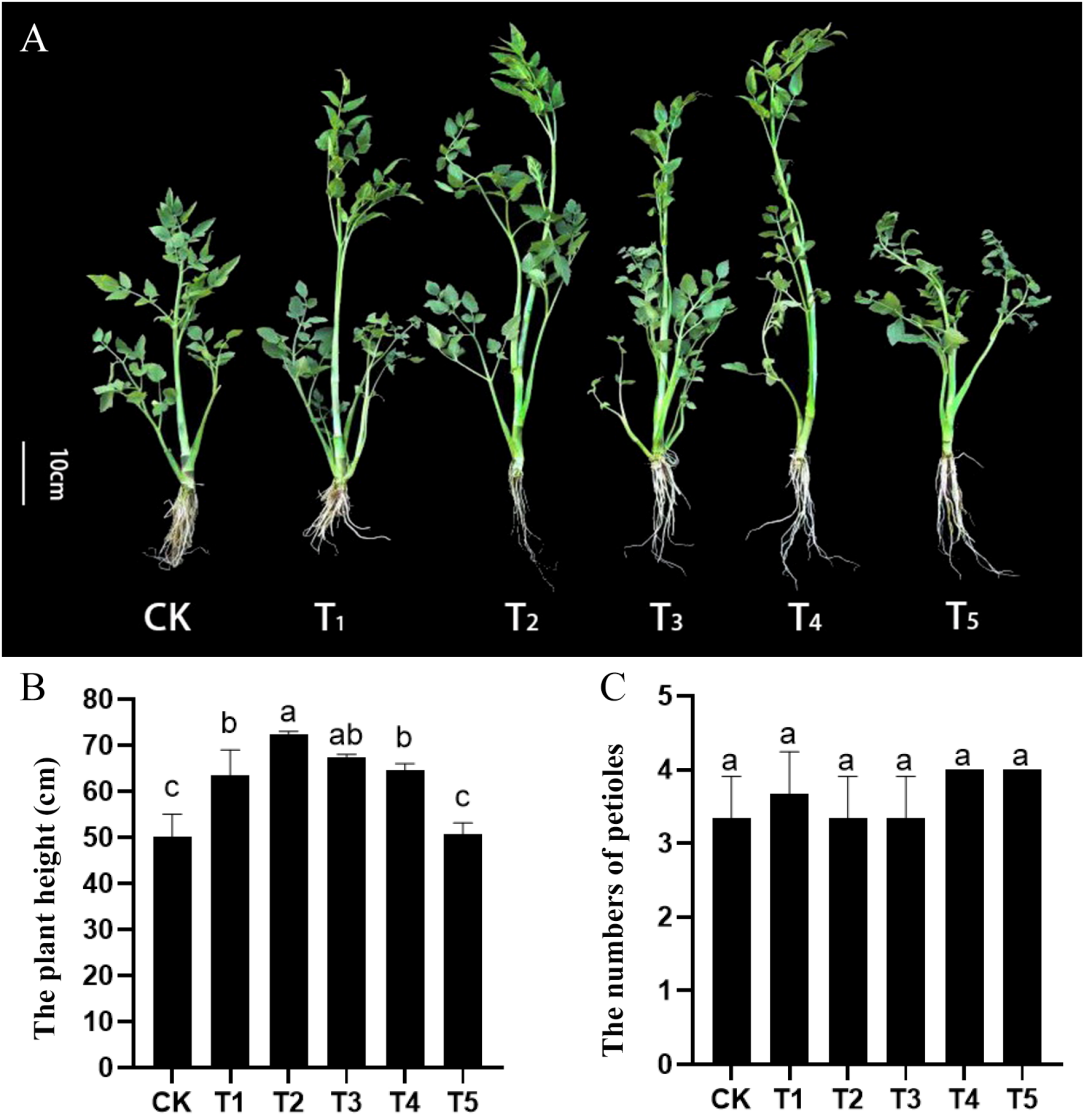
Effects of exogenous GA_3_ and uniconazole treatments on the growth of water dropwort **(A)** The growth status of water dropwort under GA_3_ and uniconazole treatments. **(B)** The plant heights under GA_3_ and uniconazole treatments. **(C)** The petioles numbers of under GA_3_ and uniconazole treatments. White lines in the left represent 10 cm in that pixel. CK, T1, T2, T3, and T4 represent 0, 40, 80, 120, 160 mg/L exogenous GA_3_ treatment, respectively. T5 represent the exogenous uniconazole (a gibberellin inhibitor) treatment. The columns with different letters indicate the significant differences at P < 0.05.

To investigate the effects of GA treatment on the growth of water dropwort, the GA_3_ with five concentrations (0, 40, 80, 120, 160 mg/L) and uniconazole (GA biosynthesis inhibitor) were used to treat water dropwort plants. The water dropwort was measured and sampled after 30 days of treatment. The growth of water dropwort was influenced by GA treatment (**Figure 1A**). Plant height of water dropwort was significantly increased after GA_3_ treatment, and the GA_3_ with concentration of 80 mg/L (T2) showed the most significant effect on the growth of water dropwort (**Figure 1B**). The effects of GA_3_ and uniconazole on the petiole numbers were not obvious (**Figure 1C**).

### 3.2 Anatomical structure analysis of water dropwort

**Figure 2:**
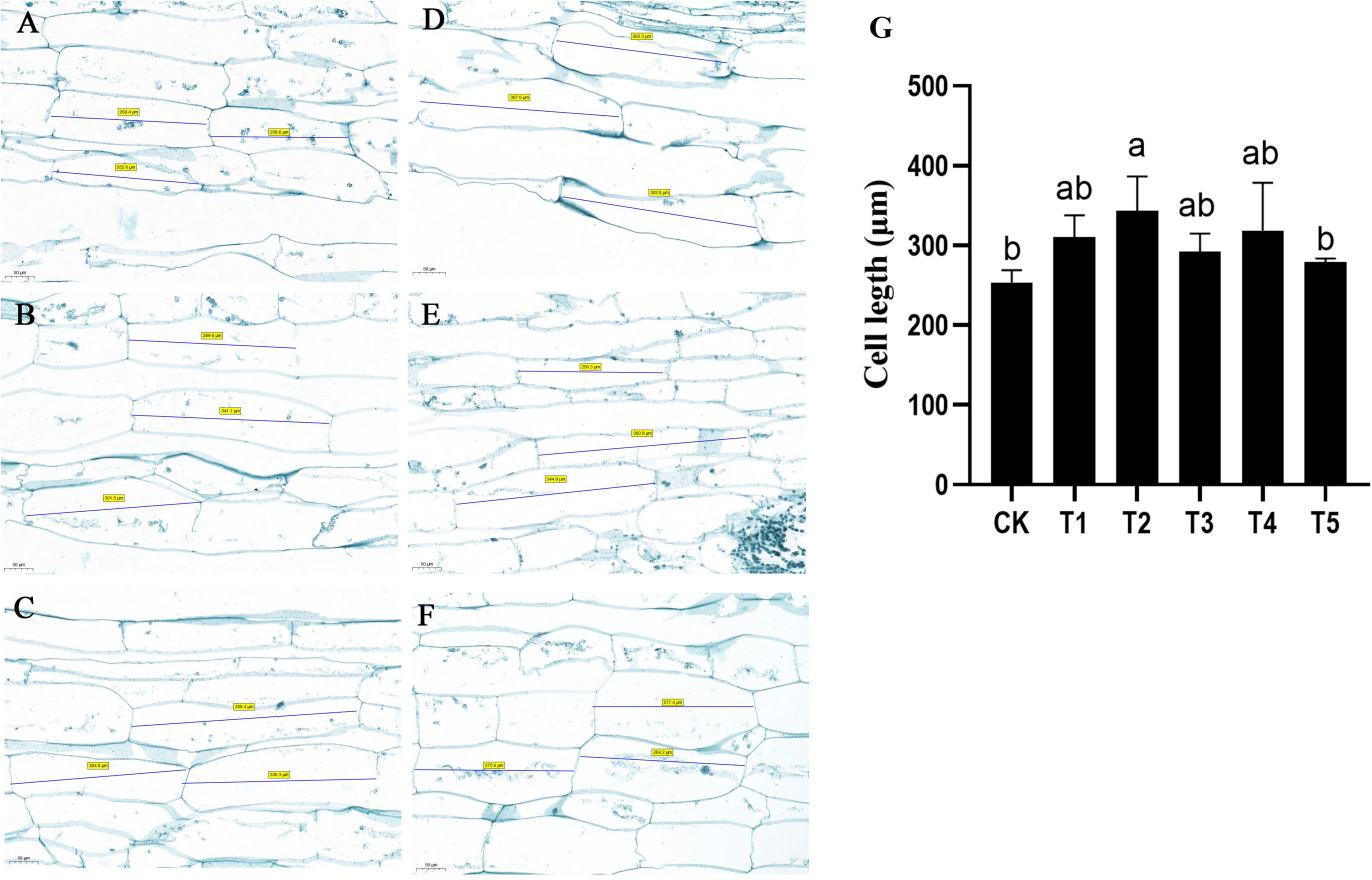
Effects of exogenous GA_3_ treatment on the cell length of water dropwort Longitudinal slice images of the water dropwort under CK **(A)**, T1 **(B)**, T2 **(C)**, T3 **(D)**, T4 **(E)**, and T5 **(F)**. The ‘number’ in the picture represents the cell length. **(G)** The statistic analysis of cell length. The columns with different letters indicate the significant differences at P < 0.05.

The anatomical structure of petioles under GA and uniconazole treatments was examined. The results indicated that the cell length of petiole was significantly increased after GA treatment (**Figures 2A–F**). The cell length of water dropwort petiole without any treatment was approximately 253.6 μm. By contrast, the cell length of petiole with 80 mg/L of GA_3_ treatment was 343.5 μm, which was significantly longer than CK and uniconazole treatments (**Figure 2G**). These results suggested that the GA-mediated increase of plant height was mainly due to cell elongation of water dropwort. Compared with other concentrations of GA treatments, the T2 (80 mg/L) treatment had the most significant promoting effect on the cell length of water dropwort.

### 3.3 Metabolites analysis of water dropwort

**Figure 3:**
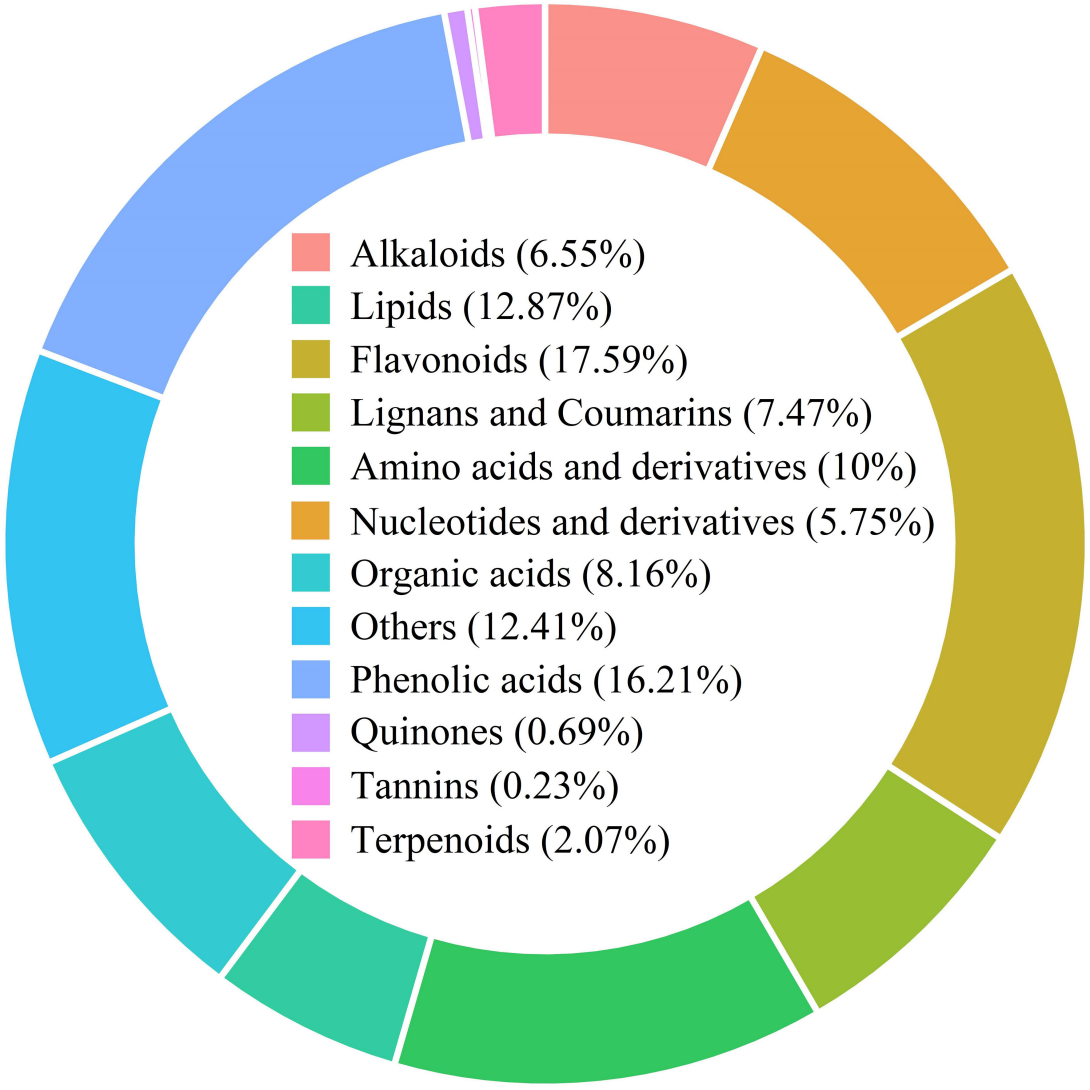
The metabolites identified from water dropwort under GA_3_ treatment based on the metabolome analysis.

The metabolomic analysis of water dropwort was performed to understand the metabolites changes under GA treatment. A total of 870 metabolites were identified from all samples of water dropwort (**Supplementary Table S1**). The detected metabolites can be classified into different categories. The flavonoids accounted for the highest proportion of 17.59%, followed by phenolic acids, amino acids and derivatives, and organic acids, which accounted for 16.21%, 10% and 8.16% respectively (**Figure 3**). Cluster analysis indicated that different metabolites accumulated in CK, T2, and T5 treatments.

### 3.4 Differentially expressed metabolites analysis

**Figure 4:**
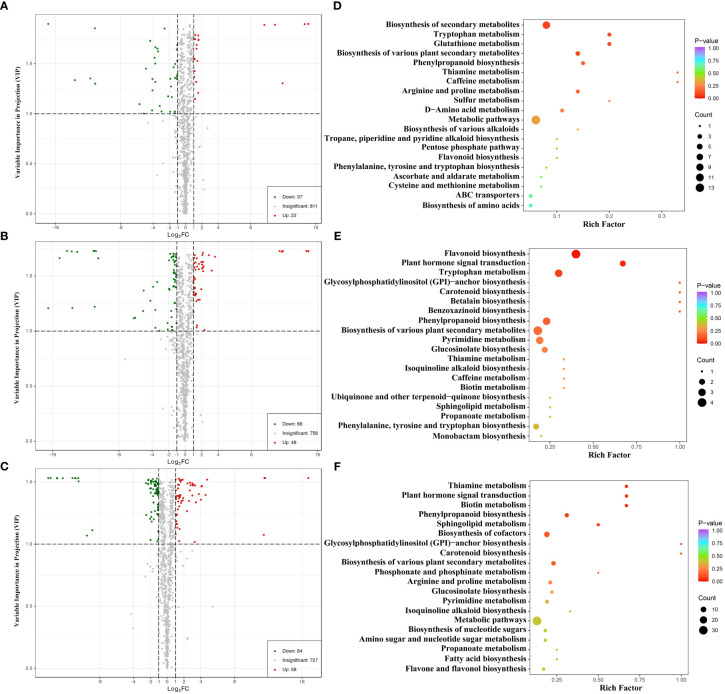
The differential expressed metabolites analysis of water dropwort under GA_3_ treatment **(A–C)**: Volcanic map of differential metabolite analysis in CK, T2, and T5 groups. **(D–F)**: The enrichment analysis of differential metabolite in CK, T2, and T5 groups.

The differentially expressed metabolites (DEMs) among CK, T2, and T5 groups of water dropwort were analyzed (**Supplementary Table S2**). The VIP based on the OPLS-DA model and FC values were combined to identify the DEMs in different groups of water dropwort (**Figures 4A–C**). The results indicated that 59 DEMs were identified in CK and T2 group, including 37 down-regulated and 22 up-regulated DEMs. A total of 114 DEMs were identified in CK and T5 group, including 66 down-regulated and 48 up-regulated DEMs. A total of 142 DEMs were identified in CK and T5 group, including 84 down-regulated and 58 up-regulated DEMs. The KEGG enrichment analysis of DEMs in different groups of water dropwort was also conducted. The results indicated that the metabolic pathways and biosynthesis of secondary metabolites were the most abundant pathways (**Figures 4D–F**).

### 3.5 Transcriptomic analysis of water dropwort

**Figure 5:**
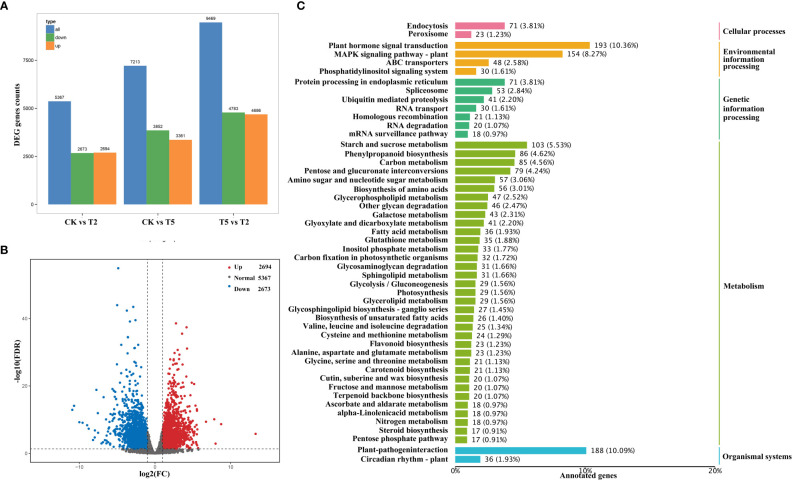
The transcriptome analysis of water dropwort under GA_3_ treatment **(A)** Statistical analysis of differentially expressed genes. **(B)** Volcanic map of differentially expressed genes analysis. **(C)** The KEGG enrichment analysis of differentially expressed genes in water dropwort.

To identify the gene expression profiles under exogenous GA treatment, the transcriptome analysis of water dropwort was conducted. A total of 63.98 Gb clean data were obtained and the percentage of Q30 bases of each sample was no less than 92.52% (**Supplementary Table S3**). The mapped efficiency of different samples with reference genome ranged from 91.20% to 94.46%. The differential expressed analysis of water dropwort under different treatments was performed (**Figure 5A**). The volcano plot indicated that a total of 5,367 DEGs were identified between CK and T2 treatments, including 2,694 up-regulated DEGs and 2,673 down-regulated DEGs (**Figure 5B**).

The annotation and enrichment analysis of DEGs was conducted according to the GO and KEGG databases. The GO enrichment analysis mainly includes the biological process, cellular component, and molecular function. As for biological process branch, the ‘carbohydrate metabolic process’ had the most abundant DEGs after exogenous GA treatment. The components involved in cell wall were also identified in the biological process branch, such as ‘cell wall organization’ and ‘cell wall biogenesis’ (**Supplementary Figure S1**). In cellular branch, the plasma membrane had the largest number of DEGs in all components. In addition, the cell wall-related components were also detected from the branch. The exogenous GA_3_ may promote plant elongation by regulating cell-related life processes in water dropwort. KEGG annotation and enrichment analysis were conducted to identify the metabolic pathways of water dropwort (**Figure 5C**). The DEGs were most enriched in the ‘plant hormone signal transduction’ pathway after exogenous GA treatment. This suggested that the exogenous GA treatment could regulate plant growth by affecting various hormone signaling pathways. In addition, the DEGs were also abundantly enriched in many metabolisms, including ‘starch and sucrose metabolism’, ‘phenylpropanoid biosynthesis’, and ‘carbon metabolism’.

### 3.6 The GAs contents and DEGs related to GA biosynthetic pathway

**Figure 6:**
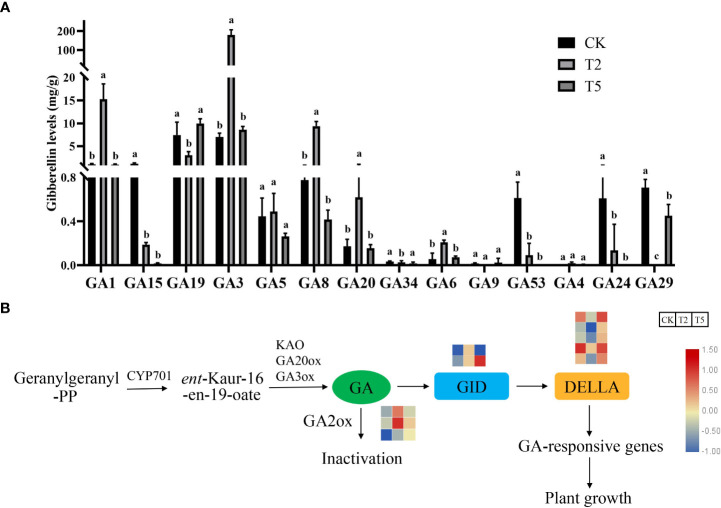
Effects of exogenous GA_3_ treatment on the various gibberellin contents and the gene expressions **(A)** The contents of different kinds of GA under GA_3_ treatment. **(B)** The expression of differentially expressed genes in GA biosynthesis and signaling transduction pathway. The columns with different letters indicate the significant differences at P < 0.05.

To investigate the effect of exogenous GA_3_ treatment on different kinds of GA, the GAs contents of CK, T2, and T5 were determined. Compared with CK and T5 treatments, the GA_3_ content under T2 treatment was significantly increased after exogenous GA_3_ treatment. In addition, the contents of GA_1_, GA_4_, and GA_8_ were also increased after GA_3_ treatment. However, some GAs were significantly decreased after GA_3_ treatment, such as GA_15_, GA_19_, GA_24_, GA_29_, and GA_53_ (**Figure 6A**). The transcriptomic analysis indicated that the GA biosynthesis and signaling transduction was significantly influenced by the exogenous GA treatment. GAs is synthesized from the geranylgeranyl-PP (GGPP). The expression trends of GA biosynthesis genes (*KAO*, *GA20ox*, *GA3ox*) in different groups varied. The transcripts of GA metabolism-related gene (*GA2ox*) were up-regulated after exogenous GA treatment. The active GAs can bind with GID proteins to form a complex in plants. The *GID* genes were up-regulated and *DELLA* genes were down-regulated after exogenous GA treatment (**Figure 6B**).

### 3.7 Identification of cellulose synthase family in water dropwort

**Figure 7:**
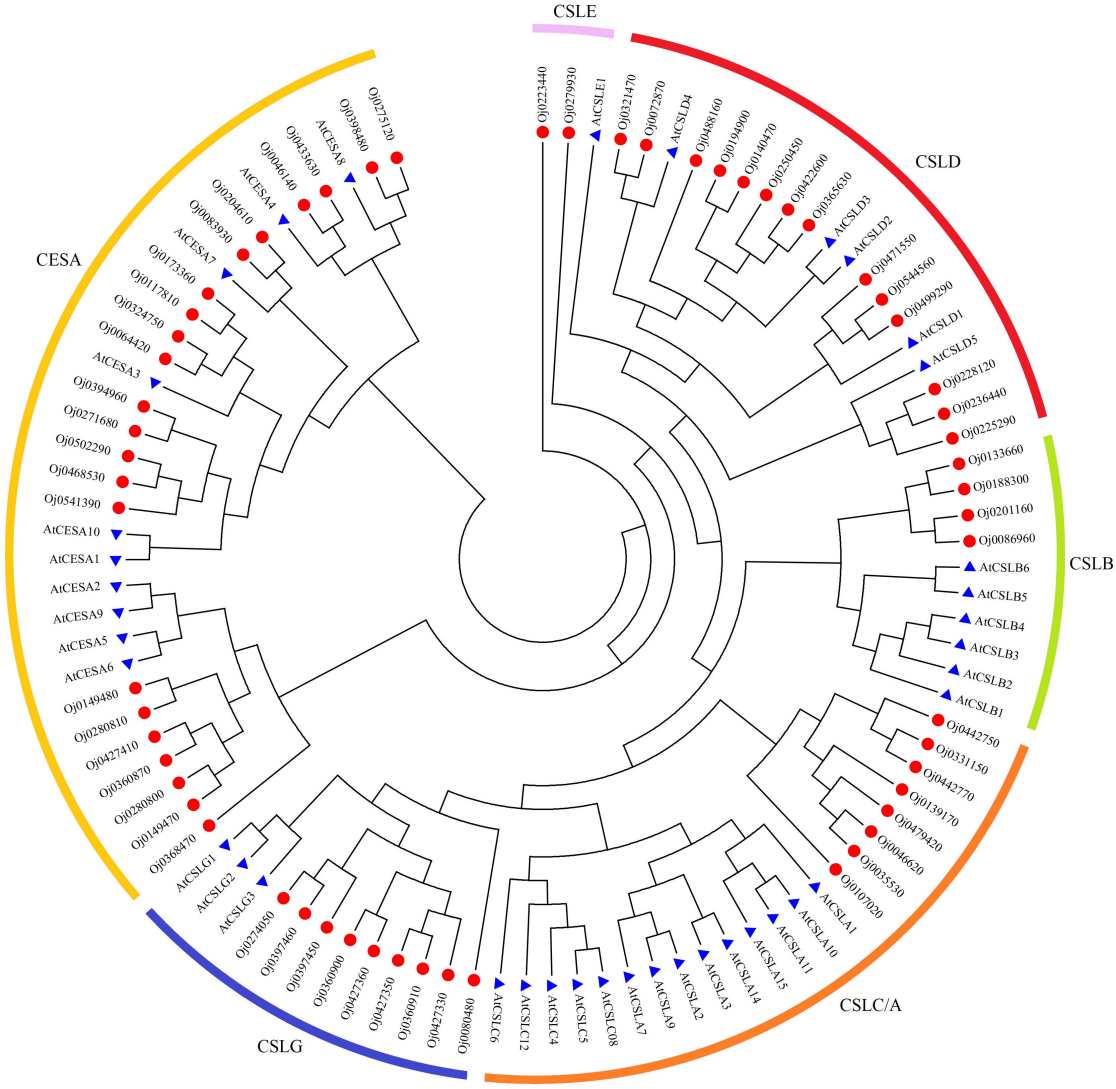
Phylogenetic analysis of cellulose synthase genes family from Arabidopsis and water dropwort.

The biosynthesis of cellulose was related to the cell wall formation in plants. A total of 59 cellulose synthase (CES) family members were identified from the genome of water dropwort. Based on the phylogenetic relationships with Arabidopsis, the CES proteins of water dropwort can be divided into different cellulose subfamilies, including synthase (CESA) and cellulose synthase-like (CSLA/B/C/D/E/G) subfamilies (**Figure 7**).

**Figure 8:**
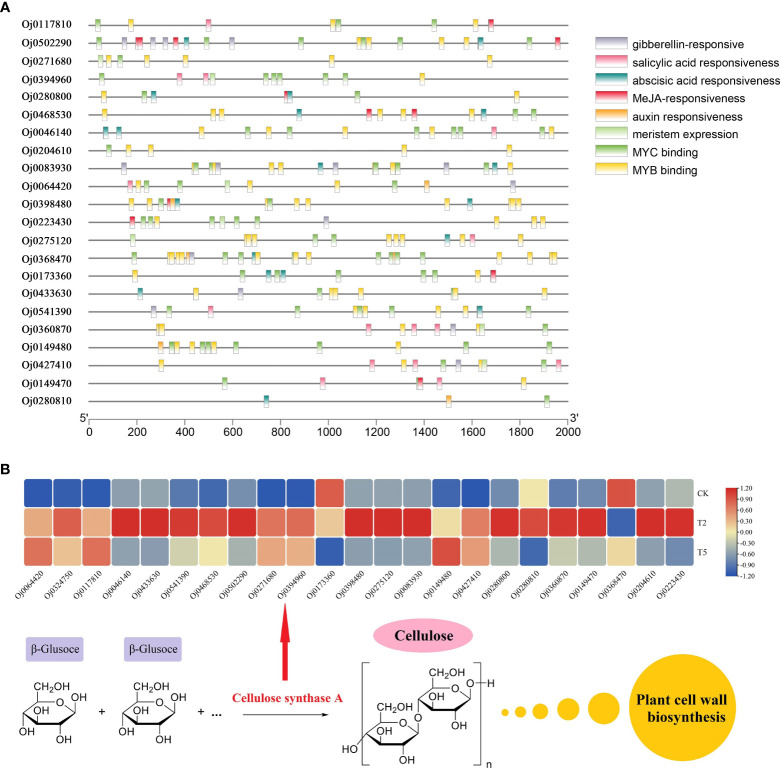
The cis-responsive elements and expression analysis of CESA family genes **(A)** The cis-responsive elements analysis of the promoters of CESA family genes. **(B)** The expressions of CESA family genes under CK, T2, and T5 treatments.

A total of 22 members were detected from CESA subfamily, and the *cis*-acting elements analysis were conducted by PlantCARE (**Figure 8A**). The gibberellin-responsive elements were discovered in the promoters of CESA family genes, suggesting these *CESA* genes may be regulated by the gibberellin signal in water dropwort. In addition, the *CESA* genes also contained abundant other hormone-related elements, such as auxin responsiveness elements, MeJA responsiveness elements, and abscisic acid responsiveness elements. The biosynthesis of cellulose was catalyzed by the CESA proteins, the transcriptions of *CESA* genes were analyzed in water dropwort (**Figure 8B**). The results indicated that most *CESA* genes were up-regulated under GA_3_ treatment. The changes of gene expression promoted the biosynthesis of cellulose, which further provided the materials for the cell wall in the cell elongation of water dropwort under GA_3_ treatment.

## 4 Discussion

Water dropwort was a perennial aquatic herb in the Apiaceae family, which is consumed as vegetable in China (Feng et al., 2018). Currently, research on water dropwort mainly focus on the regulation of flavonoids and the flavor-related substances (Kumar et al., 2021; Kumar et al., 2022; Liu et al., 2023). The published high-quality genome sequence also provided basis for the fundamental and applied research of water dropwort (Liu et al., 2021; Liu et al., 2023). Petiole and stem were the main editable parts of water dropwort. Trying to improve the yield of product organs has become a farming goal in production of water dropwort. Regulation of plant growth by plant hormones is an effective approach in agronomic production (Cai et al., 2014; Sakata et al., 2014). However, the studies aiming at using plant hormones to promote the plant height of water dropwort were still unavailable.

In the current study, the plants of water dropwort were treated with different concentrations of GA_3_ and uniconazole (a GAs inhibitor). The plant height was significantly increased after exogenous GA3 treatment, which indicated that the GAs mainly affected petiole length, but did not affect petiole number. Anatomical structure analysis indicated that the cell length was increased under T2 treatment. The reason for the lack of difference in cells length between CK and T3, T4, and T5 is mainly due to the fact that the promoting roles of GAs is concentration-dependent. GAs is a common plant hormone first identified from fungus *Gibberella fujikuroi*, which was investigated to promote the stem elongation in many plants (Eriksson et al., 2000; Claeys et al., 2014). The cell length in the second internode of *Neolamarckia cadamba* was also significantly increased after exogenous application of GAs (Li et al., 2022). The GA-deficient mutant with dwarf phenotype further proved the roles of GAs in regulating the plant height (Ashikari et al., 1999; Sakamoto et al., 2004). Our results indicated that the GA_3_ treatment promoted the petiole length by inducing the cell elongation of water dropwort.

To further investigate the changes of metabolites and gene expression under GAs treatment, the metabolome and transcriptome analysis of water dropwort was conducted. Flavonoids were the most abundant metabolites in water dropwort and the metabolome analysis indicated that the DEMs were mainly enriched in the metabolic pathways and biosynthesis of secondary metabolites under GA treatments. The accumulation of flavonoids plays an important role in the medicinal efficacy of water dropwort. The flavonoids extracted from water dropwort have many medicinal functions, such as delay the senescence (Moon et al., 2009), anti-inflammatory (Ahn and Lee, 2017; Wei and Ma, 2021), antioxidant (Seo et al., 2016), and antidiabetic effect (Yang et al., 2000).

The contents of various GAs were also increased after exogenous GA_3_ treatment. The transcriptome analysis indicated that the *GA2ox* genes were up-regulated after exogenous GA_3_ treatment, which inactivates GA (Wuddineh et al., 2015). This is due to the high levels of GAs resulted in the feedback regulation of GAs metabolism genes in plants (Gallego-Giraldo et al., 2008). The GA-mediated regulation in plants usually involves in the cooperative regulation of various plant hormones (Que et al., 2018). In this study, the transcriptome analysis indicated that numerous DEGs were significantly enriched in the plant hormone signal transduction pathway. These results indicated that GAs promoted plant elongation through synergistic regulation of multiple plant hormone signal transduction in plants. Changes of cell wall growth play an important role in the elongation process of plants (Munekata et al., 2022; Petrova et al., 2022). GO enrichment analysis showed that the exogenous GA_3_ promoted plant elongation by regulating cell-related life processes in water dropwort, such as ‘cell wall organization’ and ‘cell wall biogenesis’.

**Figure 9:**
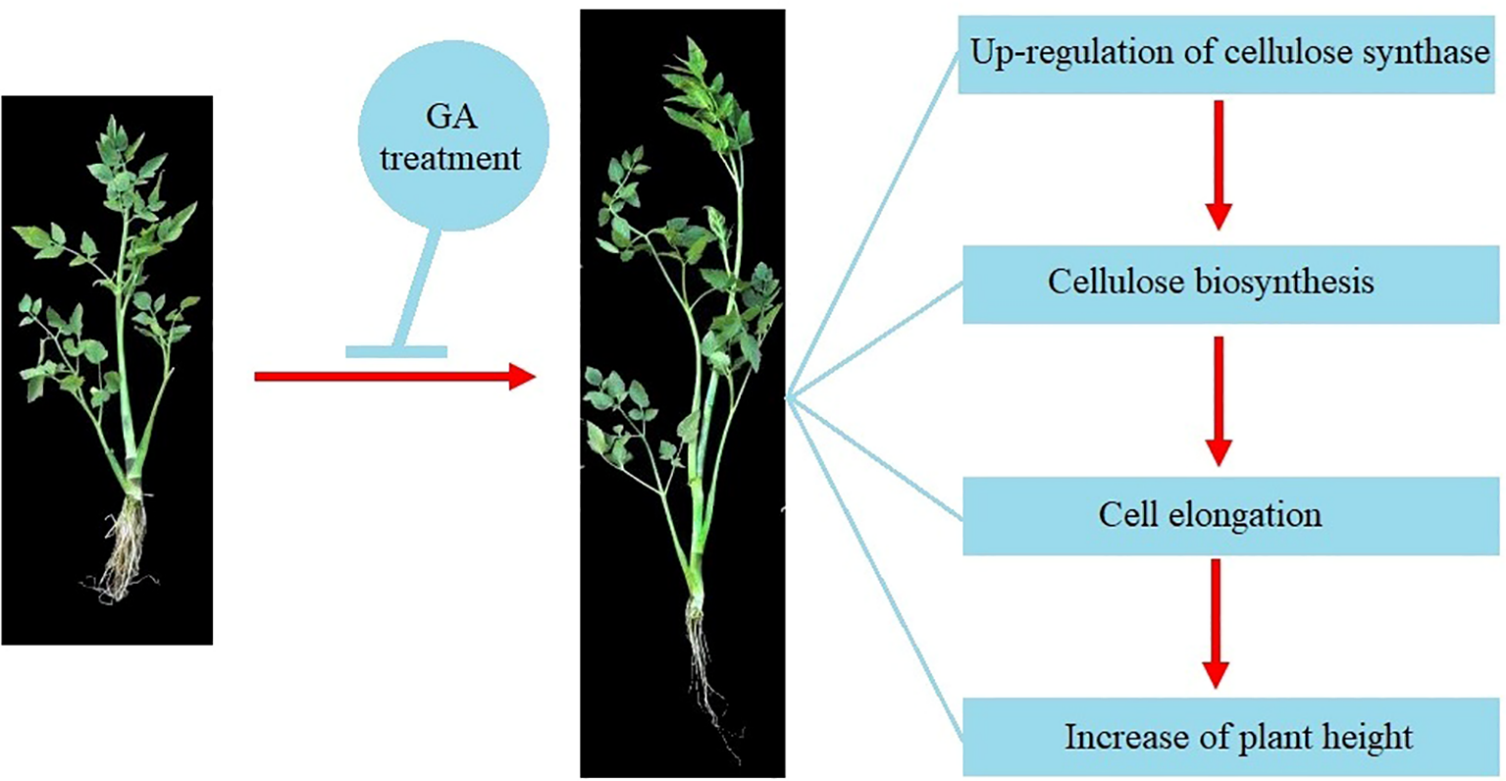
The effects of GA_3_ treatment on the growth of water dropwort.

Cellulose, a natural polysaccharide, is the main component of plant cell walls (Zhong et al., 2019). The biosynthesis of cellulose was catalyzed by cellulose synthase complex (CSC) in plants (Somerville, 2006). The cellulose synthase gene family (CES) in plants is a multi-gene family and 10 *CESA* genes were found in the Arabidopsis genome (Richmond and Somerville, 2000). The expression of *CESA* genes in plants can be regulated by different phytohormones-mediated signaling to precipitate the cell wall formation (Huang et al., 2015). In the current study, a total of 22 CESA members was identified from the genome of water dropwort. Multiple hormone-related elements including gibberellin-responsive were discovered from the promoters of *CESA* genes. Transcriptome analysis showed that the expressions of *CESA* genes were significantly up-regulated in water dropwort after GA_3_ treatment. These results suggested that the exogenous GA_3_ treatment can promote the biosynthesis of cellulose and thereby elongate cells, leading to an increase in plant height (**Figure 9**). The function of the identified key genes will be our research focus in future work.

## 5 Conclusion

In the current study, the comprehensive analysis of morphological, metabolome, and transcriptome revealed the effects of exogenous gibberellin on water dropwort. The plant height of water dropwort was significantly increased after exogenous gibberellin treatment. The metabolites and different kinds of GAs were also regulated by exogenous gibberellin treatment. These results provided valuable information for understanding the molecular mechanism of GA-mediated cell elongation in plants. This study also offered a strategy to modulate the growth by using exogenous GAs in the production of water dropwort.

## Data availability statement

The datasets presented in this study can be found in online repositories. The names of the repository/repositories and accession number(s) can be found below: NCBI BioProject https://www.ncbi.nlm.nih.gov/bioproject/, PRJNA977200.

## Author contributions

KF and LL initiated and designed the research. KF, XL, YY, RL, ZL, NS, ZY, SZ, and PW performed the experiments. KF, XL, YY, ZL, and RL analyzed the data. KF and LL contributed reagents/materials/analysis tools. KF wrote the manuscript. LL revised the manuscript. All authors contributed to the article and approved the submitted version.
