# Supplementary figures and images for: Integrated morphological, metabolome, and transcriptome analyses revealed the mechanism of exogenous gibberellin promoting petiole elongation in Oenanthe javanica
Source: Front Plant Sci. 2023 Jul 17;14:1225635. doi: 10.3389/fpls.2023.1225635 (PMC10389089; doi:10.3389/fpls.2023.1225635)

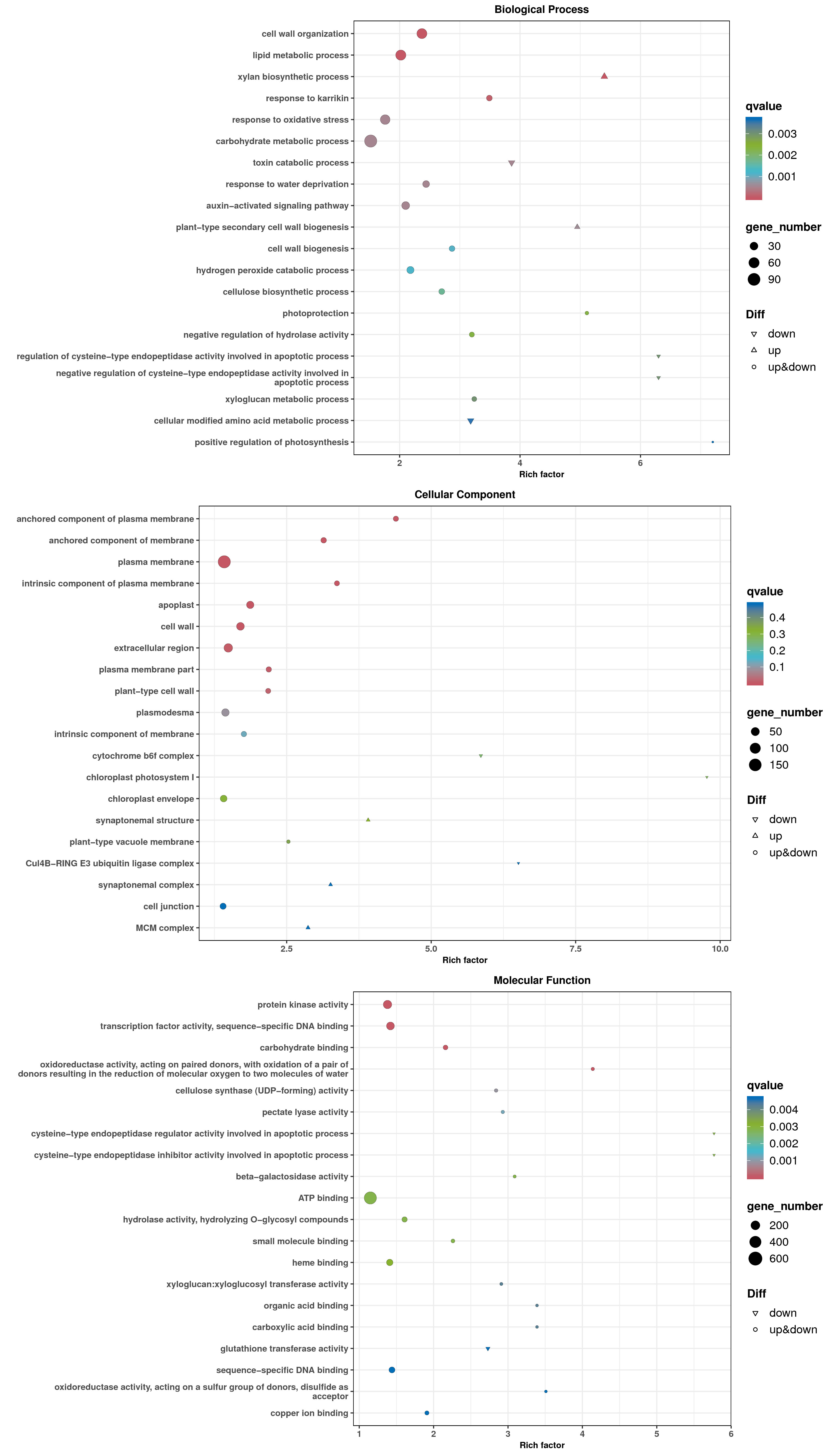

Supplement: Supplementary file 1 [file Image_1.jpeg]
